# NLRP3 Regulates Mandibular Healing through Interaction with UCHL5 in MSCs

**DOI:** 10.7150/ijbs.78174

**Published:** 2023-01-16

**Authors:** Wenhua Zhao, Yanan Cao, Yue Chen, Yuyi Chen, Tianxiao Wang, Lu Li, Yan Xu, Hua Yuan, Hua Wang, Ruixia Wang, Wen Sun

**Affiliations:** 1 Department of Basic Science of Stomatology, The Affiliated Stomatological Hospital of Nanjing Medical University, Nanjing, China; Jiangsu Province Key Laboratory of Oral Diseases, Nanjing, China; Jiangsu Province Engineering Research Center of Stomatological Translational Medicine, Nanjing, China.; 2 Department of Dental Implantology, Affiliated Hospital of Stomatology, Nanjing Medical University, Nanjing, China; Jiangsu Province Key Laboratory of Oral Diseases, Nanjing, China; Jiangsu Province Engineering Research Center of Stomatological Translational Medicine, Nanjing, China.

**Keywords:** Deubiquitination, mandibular healing, mesenchymal stem cells, NLRP3, UCHL5

## Abstract

NLRP3 has been involved in several physiological and pathological processes. However, the role and mechanism of NLRP3 activation in mandibular healing remain unclear. Here, a full-thickness mandibular defect model by osteotomy was established in wild-type (WT) and Prx1-Cre/ROSA^nTnG^ mice to demonstrate the NLRP3 inflammasome activation in mandibular healing. We found that NLRP3 was activated in mesenchymal stem cells (MSCs)-mediated mandibular healing and was prominent in Prx1^+^ cells. Inhibition of NLRP3 exerted a positive effect on bone formation without changing the number of Prx1-cre^+^ cells in the defect areas. In addition, NLRP3 deficiency promoted osteoblast differentiation. We next screened for the deubiquitinating enzymes that were previously reported to be associated with NLRP3, and identified UCHL5 as a regulator of NLRP3 activation in mandibular healing. Mechanistically, NLRP3 directly bound to UCHL5 and maintained its stability through reducing ubiquitin-proteasome pathway degradation in mandibular MSCs. At last, UCHL5 inhibition enhanced osteoblast differentiation by promoting NLRP3 ubiquitination and degradation. Thus, our results provided the proof that NLRP3 acted as a negative modulator in mandibular healing and extended the current knowledge regarding posttranslational modification of NLRP3 by UCHL5.

## Introduction

Defects of maxillofacial bone can be caused by trauma, tumor, infection and developmental malformation, all of which affect functions of oral skeletal system and decrease quality of life seriously 1. It is noteworthy that the inflammatory process is crucial for tissue repair, and dysregulated inflammation is detrimental to bone healing 2, 3. Therefore, regulating the inflammatory phase of maxillofacial bone healing is of great significance to improve the repair effect and the quality of life of patients.

Inflammasomes are multi-protein complexes assembled by intracytoplasmic pattern-recognition receptors. In recent years, more attention has been paid to nucleotide binding oligomerization domain (Nod)-like receptor family pyrin domain-containing 3 (NLRP3). The latest research suggests that endogenous full-length NLRP3 forms an inactive double-ring cage, which senses diverse stimuli, such as nigericin and extracellular ATP 4. Once activated, NLRP3 nucleates the assembly of an inflammasome, leading to IL-1-family cytokine processing 5. Mounting evidence suggests that NLRP3 plays an important role in normal skeletogenesis 6, 7 and bone diseases 8, 9. The inhibition of NLRP3 inflammasome by shRNA targeting NLRP3 can enhance healing of alveolar bone defects in diabetic rats 10. Thus, the NLRP3 inflammasome might be a double-edged sword that must be tightly regulated to avoid detrimental inflammatory responses. However, little is known about the expression or function of the NLRP3 inflammasome in non-immune cells including mesenchymal stem cells.

Mesenchymal stem cells (MSCs) are multipotent cells which self-renew and differentiate into multiple lineages of mesenchymal tissues 11. In addition to their stem properties, MSCs have also been shown to possess immunoregulatory abilities 12, 13. A previous study has reported that the expression of NLRP3 and Caspase-1 was increased in human MSCs *in vitro* under lipopolysaccharide (LPS) treatment 14. Another study found that MSCs could directly promote inflammation by NLRP3 inflammasomes 15. Currently, the intracellular pathway within mandibular bone marrow MSCs (M-MSCs) responding to inflammatory stimuli is far from clarified. The assembly of the NLRP3 inflammasome relies on protein-protein interactions, which is tightly regulated by post-translational modifications (PTMs) 16. Previous studies have established that ubiquitination is essential for the regulation of inflammasome activation, which has emerged as a potential therapeutic target for NLRP3 inflammasome-associated inflammatory disorders 17. Additionally, this process is regulated by a reversible process mediated by deubiquitylating enzymes (DUBs). In the past decade, DUBs involved in PTM control of NLRP3 complex are emerging as attractive drug targets. The biological functions of only a limited number of DUBs have been characterized, including BRCC3 18, ABRO1 19, UAF1 20, UCHL5 21, 22 and so on. Together with the findings that these DUBs involve the NLRP3 inflammasome activity, we postulated that DUBs might regulate the activation of NLRP3 inflammasome in M-MSCs.

The current study seeks to elucidate the role of NLRP3 in the process of mandibular healing with the focus on the post-translational modification stage of NLRP3 in M-MSCs. Specifically, the NLRP3 inflammasome is closely involved in the bone formation process. The local injection of MCC950, a potent highly specific small molecule inhibitor of NLRP3, promotes osteoblastic differentiation by inhibiting the NLRP3 activation. We used NLRP3^KO^ and their WT littermates to compare their mandibular healing processes, and found that knockout of NLRP3 accelerates the bone formation. Mechanistically, NLRP3 directly binds to UCHL5 and maintains its stability through reducing ubiquitin-proteasome pathway degradation in M-MSCs. At last, UCHL5 inhibition enhances osteoblastic differentiation by promoting NLRP3 ubiquitination and degradation. Thus, our findings reveal a previously unappreciated role for NLRP3 in mandibular healing via direct interaction with UCHL5 in M-MSCs.

## Methods and materials

### Mice and mandibular defect model

NLRP3^KO^ mice generated in a C57BL/6J background were kindly provided by Professor Shuo Yang 23. Prx1-Cre/Rosa^nTnG^ mice were obtained by crossing Prx1-Cre male mice with Rosa^nTnG^ female mice. For mandibular osteotomy surgery, two-month-old mice were anesthetized and prepared sterilely. Full-thickness mandibular defects (0.8 mm in diameter) were created by using a micromotor drill 24. MCC950 (MCE, Cat#HY-12815) or PBS was injected to the osteotomy site (1 mg/kg) at day 0, 1 and 2 and every 2 days thereafter. All mice were bred and maintained in the SPF Laboratory Animal Center of Nanjing Medical University. All animal procedures were conducted in accordance with approved guidelines of the Committee of Nanjing Medical University for Animal Resources.

### Cell culture

C3H10T1/2 cell lines were laboratory storage cells. Mouse mandibular bone marrow MSCs (M-MSCs) were used and generated as we described previously 25. Briefly, mandibular bones were collected. Attached soft tissues and teeth, including incisors and molars, were removed from the bones. All nucleated cells from mandibular bones were obtained by digestion with 3 mg/mL collagenase type I (Sigma-Aldrich, St. Louis, MO, USA) and 4 mg/mL dispase II (Sigma-Aldrich) for 60 min at 37°C. Single-cell suspensions were obtained through 70-μm cell strainers. We then cultured the cells in α-MEM supplemented with 10% fetal bovine serum (FBS), and 1% penicillin/streptomycin (P/S). The third passage M-MSCs were primed with 10 μg/mL LPS, and then stimulated with ATP (5 mM). Cell lysates and supernatants were collected to detect NLRP3 inflammasome activation via Western blot. For CFU-F and CFU-ALP colony formation assays, mouse mandibular bone marrow cells were cultured in 12-well-plates at 1×10^6^ cells per well with or without 50 mg/mL ascorbic acid and 10mM β-glycerophosphate for 12 days. At the end of the culture period, cells were stained with Methylene blue for total colonies (CFU-F) and stained with a BCIP/NBT alkaline phosphatase color development kit (Beyotime, Cat#C3206) for ALP-positive colonies (CFU-ALP). Positive colonies (>20 cells in a single colony) were counted. For bone nodule formation, mouse mandibular bone marrow cells were cultured in α-MEM containing 10% FBS for 7 days to generate M-MSCs. M-MSCs were then cultured in OB-inducing medium for another 14 days and mineralized bone nodules were counted after Alizarin Red S staining as described 25.

### Micro-CT, histology, and histomorphometric analyses

For micro-CT, mandibles were fixed overnight in 4% paraformaldehyde, and scanned at high resolution (10.5μm) on a VivaCT40 micro-CT scanner (Scanco Medical, Bassersdorf, Zurich, Switzerland) using 300 μs integration time, 55 kVp energy, and 145 μA intensity. 3D images were generated using a constant threshold of 275 for all samples. For histology and histomorphometric analyses, mandibles were fixed in 4% paraformaldehyde, decalcified in 14% EDTA. After dehydration, the mandibles were embedded in paraffin for paraffin sections or embedded in Tissue-Tek OCT compound for frozen sections for 3 levels (20 μm apart). The paraffin sections were stained with H&E for routine histology and for ALP activity to identify osteoblasts, TRAP activity to identify osteoclasts, Alcian blue staining to identify cartilage. The frozen sections were mounted with Mounting Medium containing DAPI (Vector) and images were captured with a Leica DM4000 fluorescence microscope, as we reported previously 26.

### Immunohistochemistry and immunofluorescence staining

The deparaffinized sections were subjected to heat mediated antigen retrieval, and blocked in H_2_O_2_ for 30 minutes, followed by PBS with 5% BSA and 0.2% Triton X-100 at room temperature for 30 minutes, and then stained overnight with primary antibody against NLRP3 (Abcam, Cat#ab214185), Prx1 (Origene, Cat#TA803116), USP1 (CST, Cat#D37B4) or UCHL5 (Abcam, Cat#ab133508) at 4 °C. For immunohistochemistry staining, after rinsing with PBS for 15 minutes, tissues were incubated with HRP-conjugated secondary antibody at room temperature. Sections were then washed and colors were developed with DAB (3,3ʹ-diaminobenzidine). Next, hematoxylin was used as a counterstain. For immunofluorescence staining, tissues were incubated with goat anti-mouse FITC (Proteintech, Cat#SA00003-1) or goat anti-rabbit Cy3 (Proteintech, Cat#SA00009-2) at room temperature. Slides were mounted with Mounting Medium containing DAPI (Vector Labs, Burlingame, CA, USA). All the images were captured with a Leica DM4000 fluorescence microscope.

### Cell transfection

Cells were infected with a control lentivirus or a UCHL5 overexpression lentivirus. The UCHL5 overexpression lentivirus was designed, synthesized and sequence-verified by Genechem Biotech Inc (Shanghai, China). In addition, cells were transfected with small interfering negative control RNA (si-NC) or specific UCHL5 small interfering RNA (si-UCHL5), respectively, and harvested after 48 h transfection. Small interfering RNA (siRNA) was purchased from Gene Pharma Co (China) and lipofectamine 2000 (Invitrogen) was used to transfect. All of the transfection procedures followed the protocols of the manufacturer.

### Immunocytochemistry

Cells were fixed in 4% paraformaldehyde before being blocked in PBS with 10% normal goat serum and 0.1% Triton X-100 for 30 minutes, and then stained overnight with primary antibody against NLRP3 (R&D, Cat#MAB7578) and UCHL5 (Abcam, Cat#ab133508) at 4 °C. After washing with PBS for 30 minutes, cells were incubated with goat anti-rat FITC (Proteintech, Cat#SA00003-11) and goat anti-rabbit Cy3 (Proteintech, Cat#SA00009-2) at room temperature. Cells were stained with DAPI and images were captured with a Leica DM4000 fluorescence microscope.

### Western blot

Whole-cell lysates were lysed directly in RIPA buffer. Protein samples were quantitated by a BCA protein assay kit (Beyotime, Cat#P0012) and then separated by SDS-PAGE and transferred onto polyvinylidene diflouride (PVDF) membrane. Membranes were blocked in 5% milk in PBS for 3 hours at room temperature. After that, membranes were incubated at 4 °C overnight with primary antibodies against NLRP3 (Cell Signaling Technology, Cat#D4D8T), UCHL5 (Abcam, Cat#ab133508), USP1 (CST, Cat#D37B4), Ubiquitin (Proteintech, Cat#10201-2-AP), Caspase-1 (AdipoGen, Cat#AG-20B-0042), IL-1β (R&D System, Cat#AF-401-NA) and β-actin (Santa Cruz, Cat#sc-47778). The next day, after washing with PBST for 3 times, membranes were incubated with HRP-conjugated secondary antibody for 1 hour. Specific bands were developed using enhanced chemiluminescence (ECL) reagents (Tanon), visualized by Tanon-5200 Multi Chemiluminescent System (Tanon).

### Quantitative real-time PCR

Total RNA was extracted from cell cultures, or from tissues of mandibular defects with TRIzol Reagent (Invitrogen). cDNAs were reversely transcribed with the PrimeScript RT Master Mix (Takara, Cat#RR036A) and subjected for RT-qPCR using specific primers. Relative expression of mRNA was evaluated by the 2-^ΔΔCt^ method and normalized to the expression of *GAPDH*, respectively. The sequences of primer sets are shown in Supplemental Table 1.

### Image analyses and Statistical analysis

For the pixel intensity plot, the pixel brightness through a region of interest was measured using ImageJ and plotted against the X dimension. The fluorescence co-localization was analyzed via the ImageJ plugin ScatterJ 27. All data are given as mean ± SEM. Statistical analysis was performed using GraphPad Prism 8 software (GraphPad Software Inc, San Diego, CA, USA). Comparisons between 2 groups were analyzed using the 2-tailed unpaired Student's t-test. Comparisons among 3 or more groups were carried out using one-way ANOVA followed by Dunnett's post-hoc multiple comparisons. P values <0.05 were considered statistically significant.

### Study approval

All animal procedures were performed according to the approved protocol by the IACUC of Nanjing Medical University (no. 2004014). Our study design complied with ARRIVE 2.0 guidelines.

## Results

### Mandibular healing process induced by osteotomy

**Figure 1:**
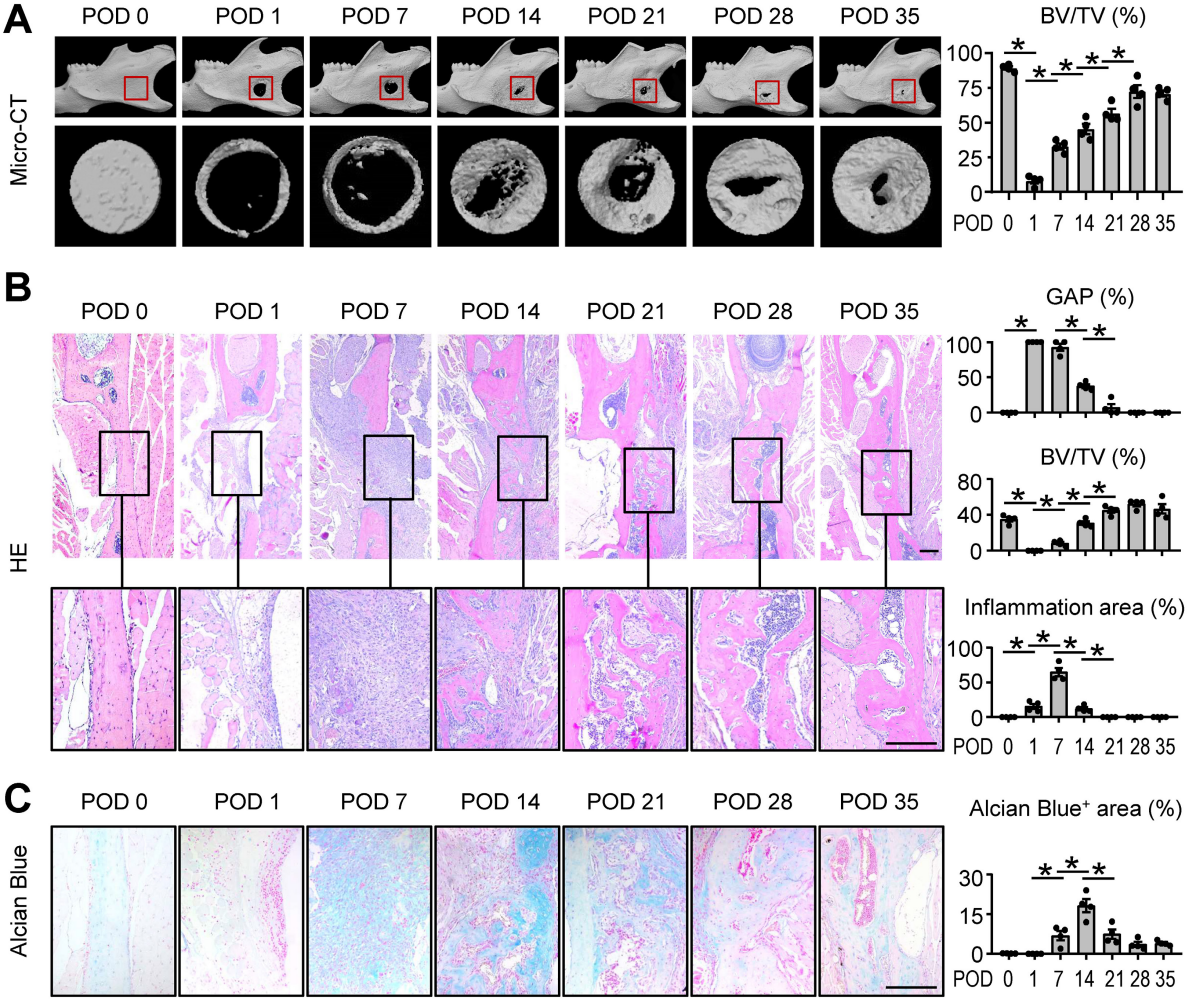
** Mandibular healing process induced by osteotomy.** Two-month-old WT mice were used. Full-thickness mandibular defects (0.8 mm in diameter) were created by using a micromotor drill. Mice were sacrificed at various time points during healing. **(A)** Representative reconstructed sections of mandibles (upper panels) and defects (lower panels) and morphometric data of bone volume (mm^3^) relative to tissue volume (%). **(B)** Representative images of H&E-stained paraffin sections show mandible defects. The gap between the ends of the defect (%), bone volume relative to tissue volume (%) and inflammation area were determined. **(C)** Alcian blue staining identifies cartilage at multiple postoperative days after osteotomy in mandible defects. Positive area (right) of blue staining was determined. Scale bars, 200 μm. All error bars represent SEM. Statistical comparisons were made using two-tailed unpaired Student's t-test. N=4, *P < 0.05 in the indicated groups.

Bone healing is a complex process which undergoes three continuing and overlapping phases: inflammation, regeneration, and remodeling 2. In order to more clearly and intuitively understand histological changes associated with mandibular healing, we established a full-thickness mandibular defect model by osteotomy in mice and performed a time-dependent histologic analysis. The mandibles were harvested at postoperative days (PODs) 0, 1, 7, 14, 21, 28, and 35. Three-dimensional reconstruction images and volumetric measurements from μCT analysis revealed that osteogenesis occurred in the defect areas of the mandibles. A five-week healing time course of the defect shows that mandibular defect areas were replaced by new bone tissues gradually, and the repair period was in the process of bone remodeling stage at PODs 28 and 35 (Figure 1A).

Histomorphometric measurements of H&E-stained tissue sections, including the gap distance between the two ends of the defect and BV/TV (%), indicated the similar results as μCT. Further, the histological staining at POD 7 suggested an active inflammatory response was occurring (Figure 1B). Alcian blue staining showed cartilage formation developed gradually and peaked at POD 14 (Figure 1C). These findings suggested the mouse model was successfully established and the typical mandibular healing process of WT mice at different phases were clearly visualized in Figure 1.

### NLRP3 inflammasome activation in MSC-mediated mandibular healing

**Figure 2:**
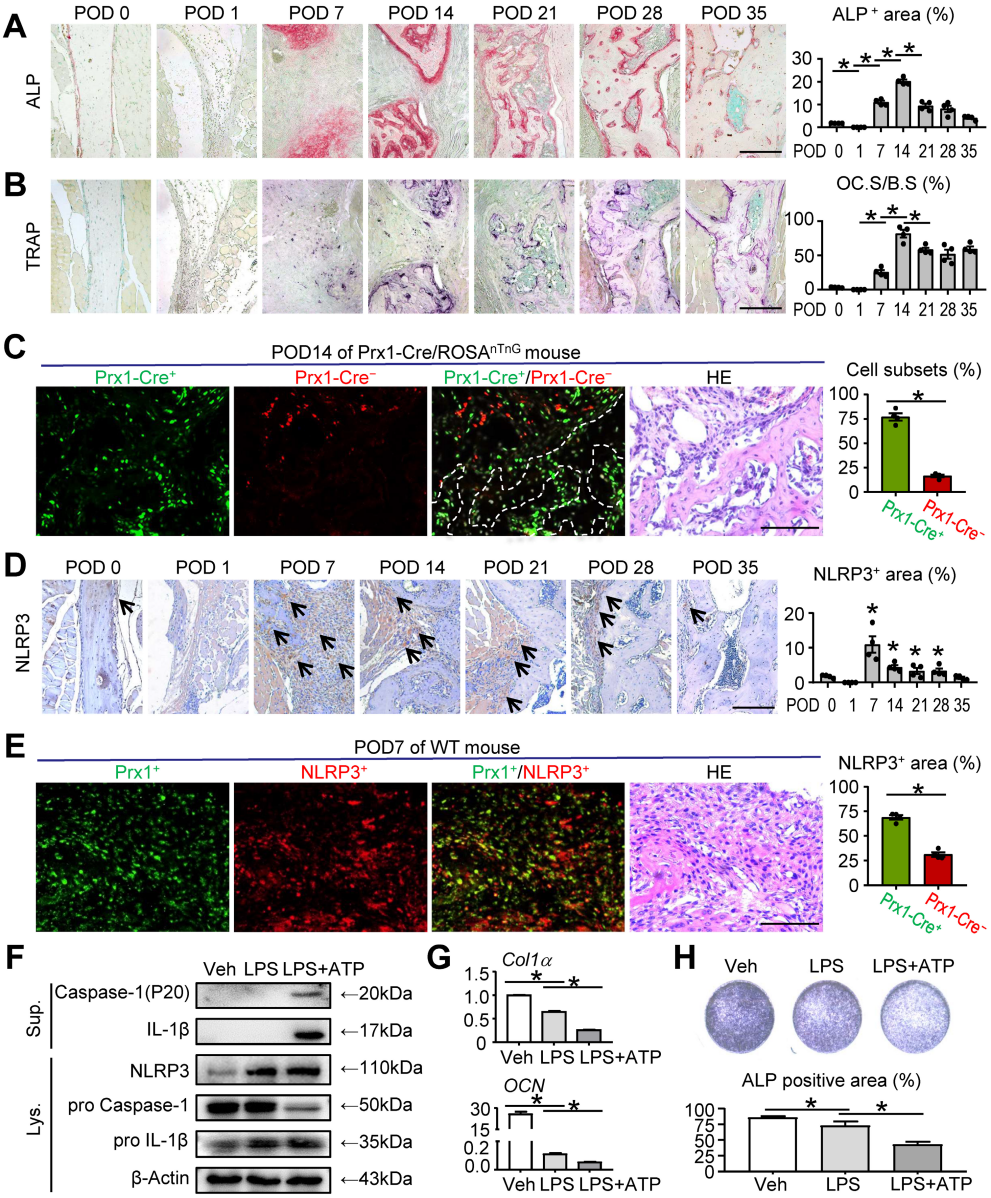
** NLRP3 inflammasome activation in MSC-mediated mandibular healing.** Two-month-old WT or Prx1-Cre/ROSA^nTnG^ mice were used, as specified in the figure legends. Mice received mandibular osteotomy surgery and were sacrificed at various time points during healing, N=4. **(A)** Representative images of ALP-stained paraffin sections show mandible defects of WT mice. ALP-positive area relative to tissue area (%) was determined. **(B)** Representative images of TRAP-stained paraffin sections show defect areas of WT mice. The surface of osteoclasts relative to the bone surface (%) was determined. **(C)** Representative images of unstained frozen sections under fluorescence microscopy and H&E-stained adjacent sections of mandible defects at POD 14 of Prx1-Cre/ROSA^nTnG^ mice. Images show Prx1-Cre^+^ cells (GFP, green) and Cre^-^ cells (tdTomato, red) in defect areas. Prx1-Cre^+^ or Cre^-^ cell number relative to all cell number (%) was assessed.** (D)** Representative IHC images for NLRP3 in defect areas at various time points. The percentage of NLRP3-positive area was calculated. **(E)** Paraffin sections of WT mice at POD 7 were subjected to double immunofluorescence staining with anti-Prx1 and anti-NLRP3. Representative images of defect areas are shown. The proportion of NLPR3^+^Prx1^+^ or NLPR3^+^Prx1^-^ area to NLRP3^+^ area (%) were determined. **(F-H)** M-MSCs from WT mice were cultured and stimulated with or without 10 μg/ml LPS ± 5 mM ATP during culture, as indicated in the figures. **(F)** Western blot of cell lysates and supernatants from M-MSCs cultures. **(G)** The relative gene expression levels of *Col1α* and *OCN* were determined by qPCR. **(H)** M-MSCs were cultured in osteoblast-inducing medium for 7 days. Culture plates were stained for ALP, and ALP^+^ areas were measured. Scale bars, 200 μm (A, B and D) and 100 μm (C and E). All error bars represent SEM. Two-tailed unpaired Student's t-test was performed. *P < 0.05 vs. POD 0 or in the indicated groups.

To gain more insight into bone regeneration stage in mandibular healing, osteoblastic bone formation and osteoclastic bone resorption were examined by ALP and TRAP staining, respectively. ALP-positive osteoblast area changes with bone healing, with obvious increase that reached a peak at POD 14, followed by a decrease at POD 21 compared with that at POD 14. A similar trend of TRAP-positive osteoclast surface was observed by TRAP staining. Both ALP-positive osteoblast area and TRAP-positive osteoclast surface were peaked at POD 14 compared with all other time points, which indicated an active bone formation status (Figures 2A and 2B). To screen for the main cell type implicated along repair course, we used Prx1-Cre/ROSA^nTnG^ mice to trace mesenchymal linage cells at POD 14. Prx1 is a marker of MSCs, which are widely used for lineage-specific knockdown or overexpression of target genes. In Prx1-Cre/Rosa^nTnG^ mouse, all Prx1-Cre^+^ cells and their descendants express GFP, whereas all Prx1-Cre^-^ cells express tdTomato 28. Notably, the sections showed that more Prx1-Cre^+^ cells were located on the surface of the trabecular bone in the defect areas compared with Prx1-Cre^-^ cells (Figure 2C), suggesting that MSCs were dominant during mandibular healing process and may exert important functions.

NLRP3 inflammasome, a central regulator of wound healing, has been reported to be closely associated with regulating bone formation 10, 29-32. Given the markedly increased level of inflammation at POD 7 (Figure 1B), which is crucial for tissue repair, we next investigated the role of NLRP3 in mandibular healing *in vivo*. We observed the expression of NLRP3 was significantly peaked at POD 7 and then declined to lower levels (Figure 2D). Double IF staining and immunohistochemical staining were performed using paraffin sections of WT mice. The results revealed that NLRP3 positive area was prominent in Prx1^+^ cells at POD 7 (Figure 2E).

To further determine the effects of NLRP3 inflammasome activation in mandibular healing, M-MSCs were exposed to LPS and ATP during osteoblast differentiation. NLRP3 inflammasome activation was confirmed by Western blot analysis, in which the expression levels of NLRP3, Caspase-1, and IL-1β were increased (Figure 2F). We next assessed the changes of osteoblast differentiation after NLRP3 inflammasome activation. The expression levels of osteoblast-related genes, including *Col1α* and *OCN*, were both significantly decreased in LPS-primed and LPS plus ATP-treated groups compared with vehicle-treated group (Figure 2G). Moreover, LPS-primed and LPS plus ATP-treated M-MSCs from WT mice formed less osteoblasts and expressed lower levels of ALP (Figure 2H). Importantly, LPS plus ATP-treated M-MSCs exhibited more activation status of NLRP3 inflammasome, formed less ALP^+^ osteoblasts, and expressed lower levels of osteoblast-related genes in the presence of ATP (Figures 2F-2H). Taken together, these data suggested that NLRP3 inflammasome plays an important role in MSC-mediated mandibular healing.

### NLRP3 deficiency promotes osteoblast differentiation and mandibular healing

**Figure 3:**
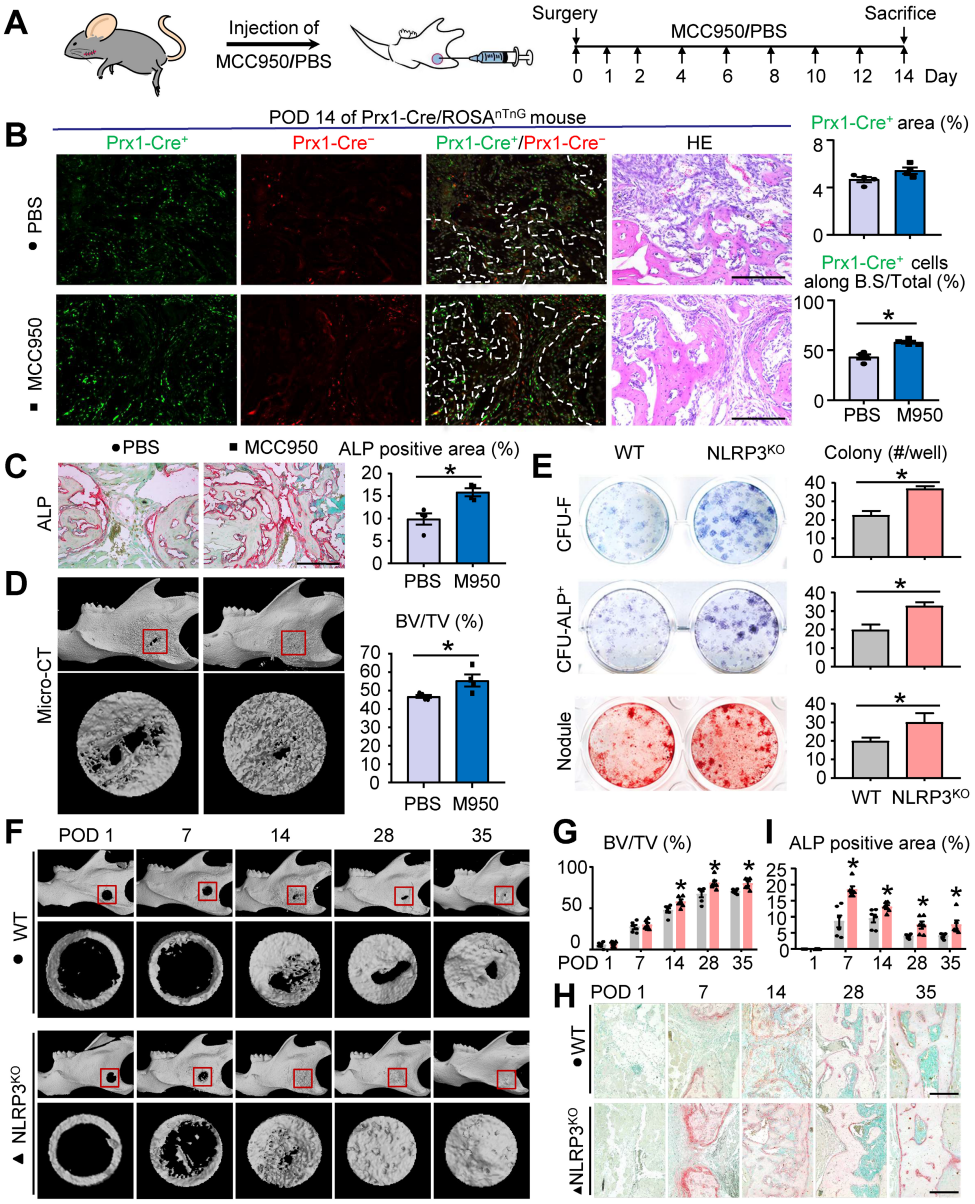
** NLRP3 deficiency promotes osteoblast differentiation and mandibular healing.** Two-month-old WT, NLRP3^KO^, or Prx1-Cre/ROSA^nTnG^ mice were used. **(A)** Schematic of experimental design. **(B-D)** Frozen sections of defect areas from Prx1-cre/Rosa^nTnG^ mice received locol injection of MCC950 or PBS were used, N=4. **(B)** Representative images of unstained frozen sections under fluorescence microscopy and H&E-stained adjacent sections of mandible defects at POD 14 of Prx1-Cre/ROSA^nTnG^ mice. Prx1-Cre^+^ area (%) was assessed. The number of Prx1-Cre^+^ cells along the bone surface relative to the number of total Prx1-Cre^+^ cells (%) was determined. **(C)** Representative images of ALP-stained frozen sections show mandible defects. ALP-positive area relative to tissue area (%) was determined.** (D)** Representative reconstructed sections of mandibles (upper panels) and defects (lower panels) and morphometric data of bone volume (mm^3^) relative to tissue volume (%). **(E)** Primary cultures of mandibular bone marrow cells from WT and NLRP3^KO^ mice were stained with methylene blue to show total CFU-F colonies, stained cytochemically for ALP to detect CFU-ALP^+^ colonies, or stained with Alizarin Red to show mineralized nodule formation. The number of CFU-F colonies, CFU-ALP^+^ colonies and nodules per well (#/well) were counted. **(F-I)** Two-month-old WT and NLRP3^KO^ mice received mandibular osteotomy surgery and were sacrificed at various time points during healing, N=6. **(F)** Representative reconstructed sections of mandibles (upper panels) and defects (lower panels). **(G)** Morphometric data of bone volume (mm^3^) relative to tissue volume (%). **(H)** Representative images of ALP-stained paraffin sections show mandible defects. **(I)** ALP-positive area relative to tissue area (%) was determined. Scale bars, 200 μm. All error bars represent SEM. Two-tailed unpaired Student's t-test was performed. *P < 0.05 in the indicated groups.

To further explore the role of NLRP3 in mandibular healing, we used MCC950 to decrease the activation of NLRP3 inflammasome *in vivo*. MCC950 is a potent highly specific small molecule inhibitor of both canonical and noncanonical activation of NLRP3 inflammasome. Two-month-old WT mice were received local injection of MCC950 or PBS after full-thickness osteotomy at PODs 0, 1, 2 and every 2 days thereafter (Figure 3A). There was no significant difference in the number of Prx1-cre^+^ cells in the defect areas between two groups. However, the number of Prx1^+^ cells along the bone surface was increased in mice receiving MCC950 than that receiving PBS (Figure 3B). Moreover, ALP-positive osteoblast area was increased in mice receiving MCC950 than that receiving PBS (Figure 3C). Three-dimensional reconstruction images and volumetric measurements from μCT analysis revealed MCC950 promoted mandibular healing (Figure 3D). These observations demonstrated that NLRP3 played an important role in osteoblastic differentiation capacity, but not the number of M-MSCs during mandibular healing.

To further confirm the effects of NLRP3 on osteoblastic differentiation, NLRP3^KO^ and their WT littermates were used for *ex vivo* and *in vivo* experiments. First, we examined CFU-F and CFU-ALP^+^ colony and nodule formation using mandibular bone marrow cells from both NLRP3^KO^ and WT mice. Cells from NLRP3^KO^ mice formed significantly increased numbers of CFU-F and CFU-ALP^+^ colonies and nodules compared to cells from WT mice (Figure 3E). In addition, expression of the osteoblastic genes including *RUNX2*, *OSX ALP*, *Col1α*, and *OCN* were up-regulated dramatically in cells from NLRP3^KO^ mice compared to that from WT mice (Figure S1). We next used mandibular defect model to verify whether NLRP3 deficiency promotes mandibular healing. As expected, micro-CT results showed greater BV/TV (%) and the accelerated bone formation and remodeling in repair period from NLRP3^KO^ mice compared with that from WT mice (Figures 3F and 3G). Moreover, ALP-positive area was increased in NLRP3^KO^ mice than that in WT mice (Figures 3H and 3I). Finally, H&E, TRAP and Alcian blue staining were also performed to evaluate mandibular healing. Consistently, the histomorphometric measurements indicated NLRP3 deficiency promoted mandibular healing process (Figure S2). Therefore, NLRP3 deficiency promotes osteoblastic differentiation and mandibular healing.

### UCHL5 is significantly increased at POD 7 in mandibular healing, which inhibits NLRP3 degradation by de-ubiquitination

**Figure 4:**
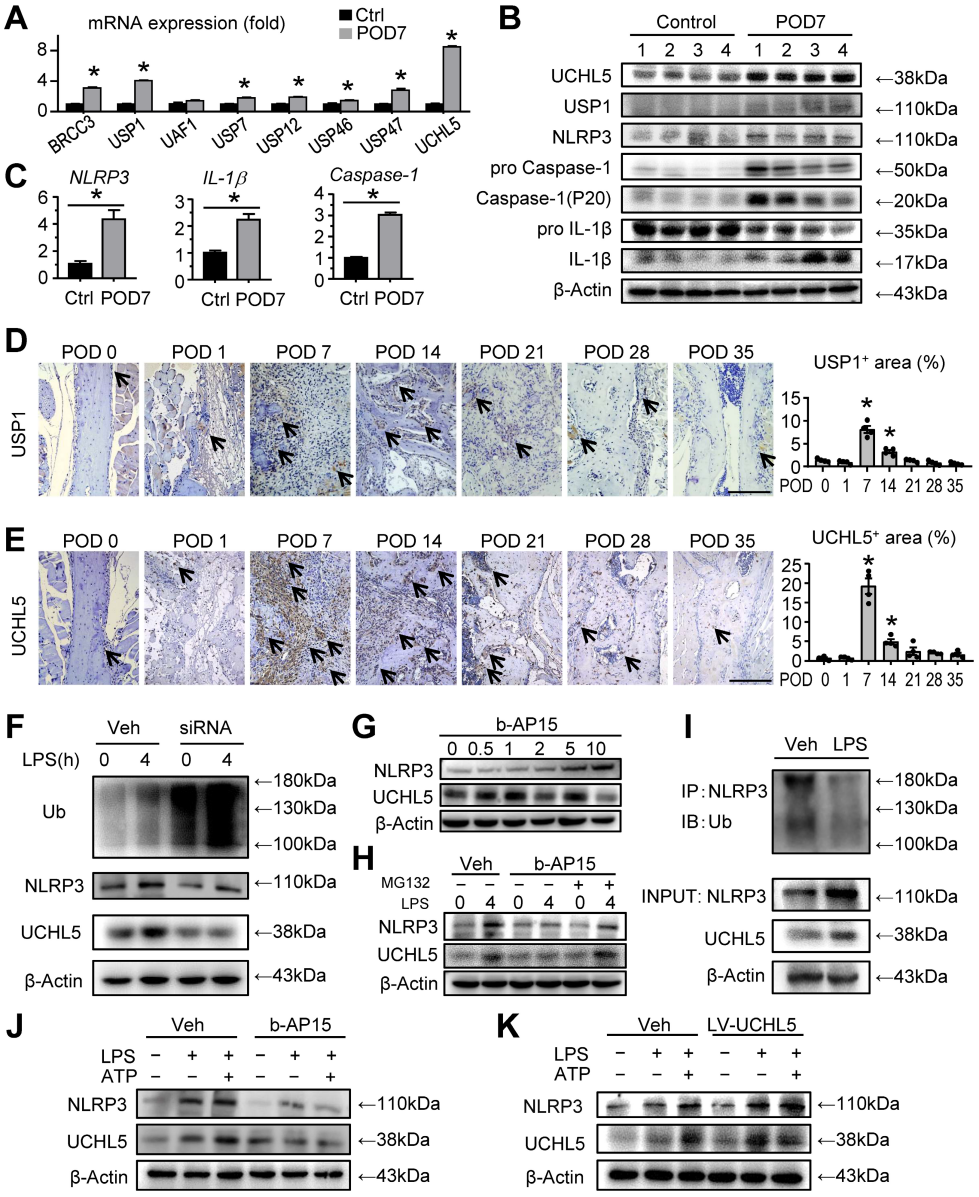
** UCHL5 is significantly increased at POD 7 in mandibular healing, which inhibits NLRP3 degradation by de-ubiquitination. (A-C)** Two-month-old WT mice received mandibular osteotomy surgery or sham surgery and were sacrificed at POD 7, N=4. **(A)** The relative gene expression levels of *BRCC3*,* USP1*,* UAF1*,* USP7*,* USP12*,* USP46*, *USP47* and *UCHL5* in tissues of mandibular defects were determined by qPCR.** (B)** Protein extracts of mandibular tissues were Western blotted to determine the expression of UCHL5, USP1, NLRP3, pro Caspase-1, Caspase-1 (P20), pro IL-1β, and IL-1β, with β-actin as the loading control. **(C)** The relative gene expression levels of *NLRP3*,* IL-1β* and* Caspase-1* in tissues of mandibular defects were determined by qPCR.** (D)** Representative IHC images for USP1 in defect areas of WT mice at various time points. The percentage of USP1-positive area was calculated. Scale bars, 200 μm.** (E)** Representative IHC images for UCHL5 in defect areas of WT mice at various time points. The percentage of UCHL5-positive area was calculated. Scale bars, 200 μm.** (F)** C3H10T1/2 cell lines were transfected with small interfering negative control RNA (si-NC) or specific UCHL5 small interfering RNA (si-UCHL5), and then stimulated with or without 10 μg/mL LPS for 4 hours. The expression levels of Ub, NLRP3, and UCHL5 were determined by Western blot analysis, with β-actin as the loading control. **(G)** C3H10T1/2 cell lines treated with different amounts of b-AP15 were detected for the optimal concentration by Western blot analysis.** (H)** Western blot analysis of NLRP3 and UCHL5 expression in C3H10T1/2 cell lines pretreated with 2 μM b-AP15 for 30 min and then stimulated with 10 μg/mL LPS for 4 h, followed by treatment with 10 μM MG132 for 1 h before harvesting the cells. **(I)** C3H10T1/2 cell lines stimulated with or without LPS were subjected to IP with anti-NLRP3 antibody and blotted with anti-Ub antibody. **(J-K)** M-MSCs from WT mice were pretreated with b-AP15 **(J)** or infected with a UCHL5 overexpression lentivirus **(K)**, and then stimulated with or without 10 μg/ml LPS ± 5 mM ATP during culture, as indicated in the figures. The expression levels of NLRP3 and UCHL5 were determined by Western blot analysis, with β-actin as the loading control. All the results are representative of at least three independent experiments. All error bars represent SEM. Two-tailed unpaired Student's t-test was performed. *P < 0.05 vs. control, POD 0 or in the indicated groups.

Since modulating inflammasome activity presents high therapeutic potential, we next set out to determine the mechanisms responsible for the activation of NLRP3 inflammasome. We screened for the DUBs that were previously reported to be associated with NLRP3, including BRCC3 18, USP1, USP12, USP46, UAF1 20, USP7, USP47 33 and UCHL5 21, 22. These DUBs, which could remove ubiquitination from NLRP3, may protect NLRP3 from ubiquitination-dependent proteasomal degradation and maintain high expression levels of NLRP3. Quantitative real-time PCR (RT-qPCR) revealed that UCHL5, together with USP1, were the top two highly expressed genes in the tissues of mandibular defects at POD 7 (Figure 4A). To further evaluate the functional DUB that targets the de-ubiquitination of NLRP3 in bone healing, we used Western blot to examine the expression levels of UCHL5 and USP1 in the tissues of mandibular defects. Compared to a moderate increase of USP1, UCHL5 was significantly increased at POD 7 (Figure 4B). In addition, the NLRP3 inflammasome activation was also evaluated for further confirmation (Figures 4B and 4C). We next examined the expression of UCHL5 and USP1 using paraffin-embedded sections by immunohistochemical staining. Similar to the observation on NLRP3, the expression of them showed similar trends (Figures 2D, 4D and 4E). While UCHL5 expression showed drastic changes, the changes of USP1 during mandibular healing were relatively modest. These data demonstrated that UCHL5 was more relevant with the inflammatory phase. Therefore, we conclude that UCHL5 might be involved in regulating deubiquitination of NLRP3 during mandibular healing.

We then cultured C3H10T1/2 cell lines and the expression of UCHL5 was silenced via small interfering RNA (siRNA), which was confirmed by RT-qPCR and Western blot (Figure S3). We observed that UCHL5 silencing significantly increased overall ubiquitylation status, however, the expression level of NLRP3 was decreased (Figure 4F). We next used b-AP15, a small molecule inhibitor that selectively inhibits the activity of UCHL5. Under a series of concentration gradient, the expression of NLRP3 increased gradually as the concentration gradient increased without any external stimuli (Figure 4G). We chose the concentration of 2 μM as optimal for the subsequent experiments. Importantly, there is a significant difference in the expression of NLRP3 between vehicle-treated group and b-AP15-treated group in response to LPS stimulation. The b-AP15-induced decrease of NLRP3 level was partially rescued by supplementation with MG132, a ubiquitination-proteasome inhibitor (Figure 4H). Consistent with the higher expressions of NLRP3 and UCHL5 upon LPS stimulation, NLRP3 ubiquitination was markedly reduced, suggesting UCHL5 inhibits NLRP3 degradation by de-ubiquitination (Figure 4I).

For further validating the connection between NLRP3 and UCHL5 in M-MSCs, we examined the expression of the indicated proteins by Western blot with respect to different interventions. As expected, the same trend of NLRP3 and UCHL5 was observed (Figures 4J and 4K). Remarkably, inhibiting UCHL5 by b-AP15 concurrently reduced NLRP3 protein levels (Figure 4J). On the other hand, increased UCHL5 expression leads to high expression of NLRP3, particularly in cells that infected with the UCHL5 overexpression lentivirus (LV-UCHL5) (Figures 4K). Taken together, these data indicate that UCHL5 is significantly increased at POD 7 in mandibular healing, which inhibits NLRP3 degradation by de-ubiquitination.

### UCHL5 interacts with NLRP3

**Figure 5:**
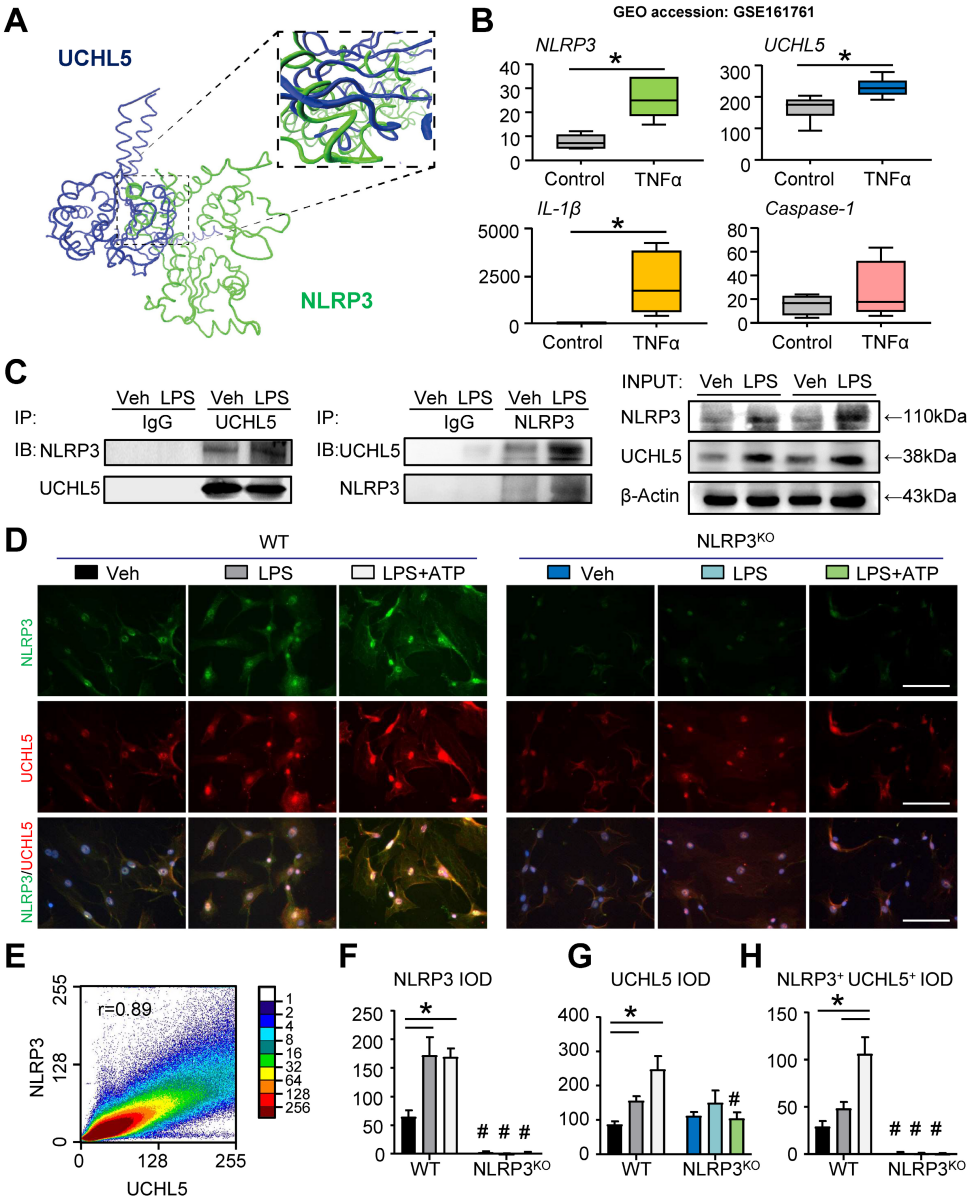
** UCHL5 interacts with NLRP3. (A)** Protein-protein docking was performed using ZDOCK. Visualization and image generation were carried out using the VMD. The blue denotes UCHL5 and green for the NLRP3. **(B)** The expressions of *NLRP3*, *UCHL5*, *IL-1β*, and *Caspase-1* in human MSCs stimulated by TNFα were determined by RNA-seq analyses. RNA-seq data were downloaded from the GEO database (GSE161761). **(C)** C3H10T1/2 cell lines were stimulated with or without 10 μg/ml LPS for 4 hours. Total cell lysates were extracted and subjected to IP with anti-UCHL5 or anti-NLRP3 antibody and blotted with anti-NLRP3 or anti-UCHL5, respectively. **(D)** WT M-MSCs were stimulated with or without 10 μg/ml LPS ± 5 mM ATP and subjected to IF with anti-NLRP3 and anti-UCHL5. Representative images are shown. Scale bars, 100 μm. **(E)** A representative 2D correlation scatter diagram of the co-localized NLRP3 and UCHL5 of WT M-MSCs stimulated with LPS plus ATP. Pearson's correlation coefficient (r = 0.89) was calculated using ImageJ software with ScatterJ analysis. **(F-H)** NLRP3^+^, UCHL5^+^ and NLRP3^+^UCHL5^+^ integrated optical densities (IODs) were determined. All error bars represent SEM. One-way ANOVA followed by Dunnett's post-hoc multiple comparisons was performed. *P < 0.05 vs. Veh or in the indicated groups, #*P* < 0.05 vs WT treated with the same reagent.

To deduce insight into the nature of molecular interactions between NLRP3 and UCHL5, we performed protein-protein docking studies. Protein domains were from UniProt and the three-dimensional structure was predicted with ZDOCK. We used molecular docking to demonstrate that NLRP3 and UCHL5 have corresponding binding sites (Figure 5A). In parallel with this approach, we downloaded and analyzed the datasets of human MSCs published previously in the GEO database (GEO accession: GSE161761). The expression of genes, including *NLRP3*, *UCHL5* and *IL-1β*, was significantly upregulated in TNFα-treated human MSCs (Figure 5B).

For further validation, we determined whether UCHL5 interacts with NLRP3. The co-immunoprecipitation (IP) assay was performed with C3H10T1/2 cell lines. Through analysis, we observed that UCHL5 could directly bind to NLRP3, and vice versa. Furthermore, LPS stimulation could promote the interaction of UCHL5 with NLRP3 (Figure 5C). We next performed immunofluorescence assays to further investigate the binding of UCHL5 to NLRP3 in M-MSCs from WT and NLRP3^KO^ mice treated with or without LPS ± ATP. When compared with vehicle-treated group, LPS-primed and LPS plus ATP-treated M-MSCs from WT mice resulted in significantly increased colocalization between NLRP3 and UCHL5 (Figures 5D and 5H), especially after stimulated with ATP (Figure 5E). On the contrary, M-MSCs from NLRP3^KO^ mice showed no significant difference between groups. The results also showed that NLRP3 and UCHL5 expression levels were increased after stimulation in the M-MSCs from WT mice, but not significantly altered in the M-MSCs from NLRP3^KO^ mice (Figures 5F and 5G). Together, these data verify that UCHL5 directly interacts with NLRP3 and induces the activation of NLRP3 inflammasomes.

### UCHL5 inhibition enhances osteoblast differentiation by promoting NLRP3 ubiquitination and degradation

**Figure 6:**
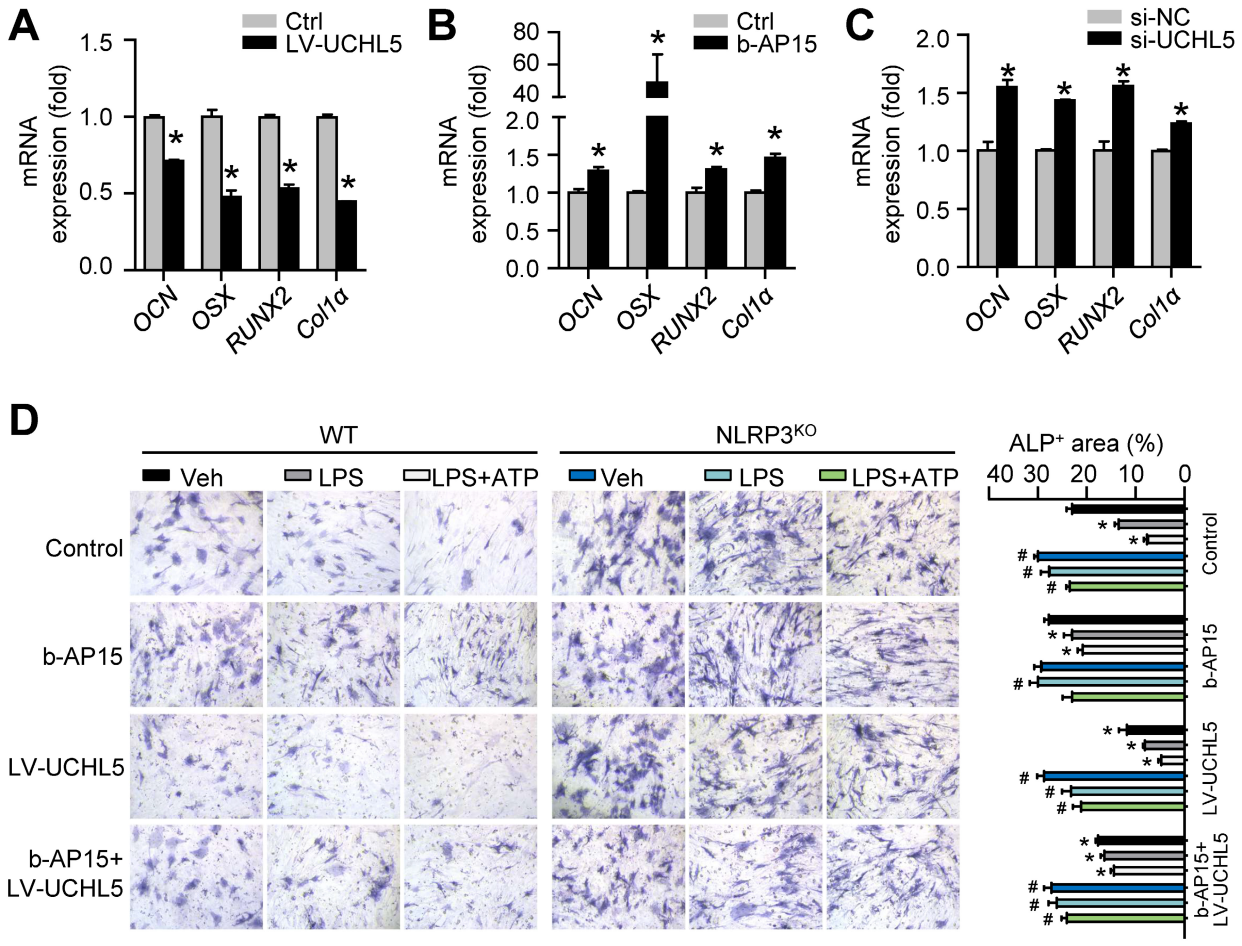
** UCHL5 inhibition enhances osteoblast differentiation by promoting NLRP3 ubiquitination and degradation.** M-MSCs from WT mice were infected with control or UCHL5 overexpression lentivirus **(A)**, treated with b-AP15 **(B)**, or transfected with si-NC/si-UCHL5** (C)**. Cells were cultured in osteoblast induction medium for 7 d.** (A-C)** The relative gene expression levels of *OCN*, *OSX*, *RUNX2* and *Col1α* were determined by qPCR. *P < 0.05 vs. control or si-NC. **(D)** M-MSCs from NLRP3^KO^ mice and their WT littermates were stimulated with or without 10 μg/ml LPS ± 5 mM ATP and subjected to ALP staining, and ALP^+^ areas were measured. *P < 0.05 vs. WT-Veh, #*P* < 0.05 vs. WT treated with the same reagent. All error bars represent SEM. One-way ANOVA followed by Dunnett's post-hoc multiple comparisons was performed.

To follow up our above findings demonstrating that inhibiting UCHL5 concurrently increased NLRP3 degradation by ubiquitination (Figures 4F-K), we then asked whether UCHL5 regulates osteoblast differentiation by preventing degradation of NLRP3. We infected M-MSCs from WT mice with UCHL5 overexpression lentivirus (LV-UCHL5). UCHL5 overexpression significantly downregulated *OCN*, *OSX*, *RUNX2* and *Col1α* mRNA levels (Figure 6A). Next, we used b-AP15 and si-UCHL5 to inhibit the expression of UCHL5. In contrast, the osteoblast-related genes expression was increased upon UCHL5 inhibition (Figures 6B and 6C).

To confirm the data, the effect of UCHL5 on osteoblast differentiation was examined using M-MSCs from NLRP3^KO^ mice and their WT littermates with osteoblast induction medium. The ALP-positive areas indicating osteogenesis were decreased in M-MSCs from WT mice in response to NLRP3 activation and increased upon UCHL5 inhibition, and the changes were blocked by NLRP3 deficiency (Figures 6D and S4). In addition, the decrease of ALP-positive area with LV-UCHL5 was partially rescued by supplementation with b-AP15. This observation became even more evident when M-MSCs in LPS-primed and LPS plus ATP-treated groups were compared. In contrast, the percentage of ALP-positive areas maintained high levels and was not significantly altered in NLRP3^KO^ mice compared with that in WT littermates (Figure 6D). These data suggest that UCHL5 inhibition enhances osteoblast differentiation by promoting NLRP3 ubiquitination and degradation.

## Discussion

**Figure 7:**
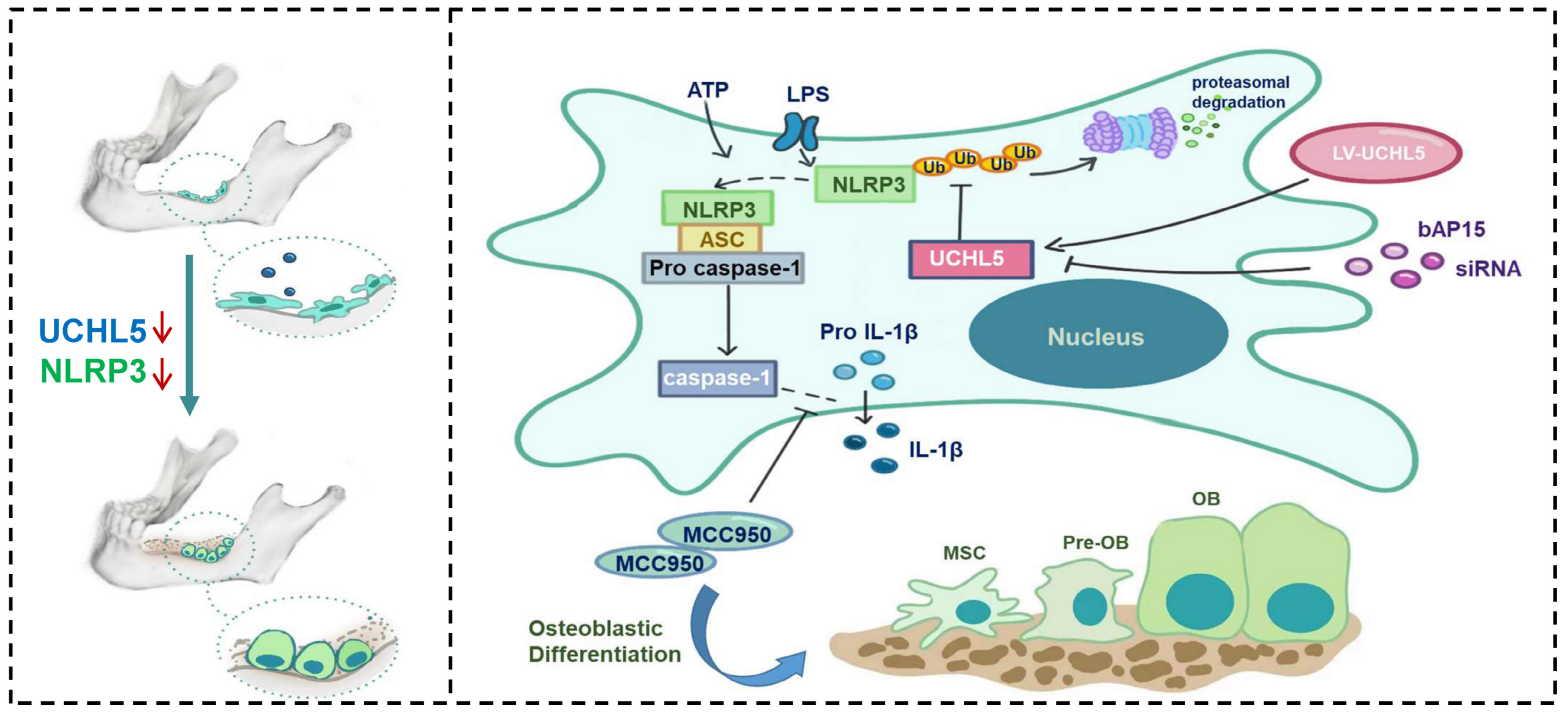
** Schematic representation of a model depicting how NLRP3 regulating mandibular healing through interaction with UCHL5 in MSCs.** During mandibular healing, NLRP3 directly binds to UCHL5 in mesenchymal stem cells, which results in a higher stability of NLRP3 through reducing ubiquitin-proteasome pathway degradation. Next, NLRP3 is activated and nucleates the assembly of an inflammasome, leading to IL-1-family cytokine processing, which inhibits osteoblast differentiation and mandibular healing. MCC950, as a potent highly specific small molecule inhibitor of NLRP3, enhances osteogenic differentiation of mandibular MSCs. Furthermore, UCHL5 inhibition exerts the same effect by promoting NLRP3 ubiquitination and degradation.

Increasing evidence suggests that NLRP3 inflammasome plays a vital role in regulating bone homeostasis 7, 29, 34, 35. Here, we demonstrated that NLRP3 deficiency specifically promotes osteoblast (OB) differentiation both *in vitro* and *in vivo*. In addition, the reduced UCHL5 expression markedly decreased the activity of the NLRP3 inflammasome. Furthermore, we indicated that UCHL5 directly interacts with NLRP3 and induces the activation of NLRP3 inflammasomes in M-MSCs, which inhibits OB differentiation by promoting NLRP3 de-ubiquitination (Figure 7). Thus, our study provides a potential molecular mechanism for the regulatory roles of NLRP3 in mandibular healing.

The fundamental understanding of tissue repair suggests that inflammation is necessary for stimulating a pro-regenerative environment to ensure optimal bone regeneration. Some studies have reported the beneficial effects of inhibition of NLRP3 on maxillary remodeling 10. Biological processes of mandibular healing involve numerous cell types, performing different functions according to its cellular localization. Previous studies shown that NLRP3 regulates alveolar bone loss in periodontitis by promoting osteoclastic differentiation 26, 31. However, by using Prx1-Cre/ROSA^nTnG^ mice, we demonstrated that there were mainly mesenchymal linage cells in the defect areas. Henceforth, we cultured mesenchymal stem cells to further explore the mechanism of NLRP3. Past research has shown that the activation of NLRP3 inflammasome leads to release of the pro-inflammatory cytokines that recruit immune cells to sites of pathogenic infection, such as IL-1β 36. It has been reported that IL-1β inhibits osteoblastic differentiation through the activation of NF-κB and MAPK signaling 37, 38. Notably, we observed the deficiency or inhibition of NLRP3 accelerated bone formation. Other mechanisms involved in the inflammatory phase might compensate for lack of NLRP3 signaling. For instance, CCL2 released from the platelets recruit monocytes and neutrophils, which release additional chemokines to recruit other cell types 39, 40. These results suggest that NLRP3 plays an active role during the initial inflammatory stage, but may be a negative modulator during regeneration and remodeling stages. To further demonstrate the double-edged effect of NLRP3, we can use tamoxifen-inducible gene recombination in the future.

MCC950, as a small-molecule inhibitor of NLRP3, is of particular interest. It has been widely verified for the treatment of multiple NLRP3 inflammasome-related disease models 41, 42. We used Prx1-Cre/ROSA^nTnG^ mice to confirm that local injection of MCC950 promotes OB differentiation. Although local injection of the drug promoted mandibular healing, the effects were incomplete. In the future, a drug delivery system of MCC950 need to be developed to ensure drug loading efficiencies and controlled release profiles, such as nanomaterials and hydrogel composites. We previously reported that MCC950 acts directly on osteoclast precursors, and thereby prevents osteoclast differentiation in periodontitis 26. In addition, MCC950 significantly decreased neutrophil extracellular trap formation (NETosis) than observed in vehicle-treated neutrophils 43. These data, together with our findings, indicated that the role of MCC950 in mandibular healing may be the result of the synergistic effect of multiple cells. Therefore, generating mesenchymal-specific NLRP3-overexpression or knockout mice by crossing NLRP3^TG^ mice or NLRP3-floxed mice with Prx1-cre mice would be beneficial for future research. It is necessary to further clarify the exact molecular events in M-MSCs, so as to fine-regulate the expression of NLRP3. We observed the changes in total ubiquitination level under LPS-induced stimulation, suggesting that the LPS-induced inflammatory response altered the post-translational modification phase of proteins in cells, which drives us to explore the post-translational modifications of NLRP3.

At present, extensive studies have demonstrated that ubiquitination is critical to the regulation of inflammasome activation and DUBs may be potential therapeutic targets for NLRP3 inflammasome-associated inflammatory disorders. NLRP3 has been reported to be ubiquitinated with K48, K63 and K27 linkages 17, 44. Thus, we profiled the expression of these reported DUBs by RT-qPCR and found that UCHL5 was highly expressed in the defect areas. UCHL5, which belongs to the ubiquitin C-terminal hydrolase (UCH) family, is well known to play an important role in the ubiquitin-proteasome system. It is involved in Wnt signaling 45, NF-κB activation 46, adipogenesis 47 and so on. In previous research, the activity of UCHL5 can be modulated by several factors, including hRpn13 and hINO80 48. A very recent study showed that de-ubiquitination of NLRP3 by UCHL5 plays an important role in the assembly and activation of inflammasome in HCV-infected hepatocytes 22. However, whether UCHL5 interacts with NLRP3 in M-MSCs remains unclear. In our study, we further confirmed that UCHL5 interacts directly with NLRP3 and predicted the associated binding sites at the molecular level. b-AP15 is a DUB inhibitor that selectively blocks deubiquitylating activity of UCHL5 and has been investigated in a few animal experiments 49, 50. In current study, we observed that M-MSCs have decreased capacity for osteoblastic differentiation after UCHL5 upregulation, which was partially corrected by b-AP15. However, b-AP15 not only regulates UCHL5, but also acts on USP14. It has been reported that USP14 may up-regulate the production of pro-inflammatory cytokines through the NF-κB pathway 51, and USP14 may enhance the dedifferentiation effect of IL-1β on chondrocytes 52. Recently, an inhibitor of UCHL5 was designed by possibly blocking its deubiquitinating function, and contributed to the accumulation of polyubiquitinated substrates 53. In order to further confirm the main role of UCHL5 in mandibular healing, the development of specific inhibitors targeting only UCHL5 might be needed in future studies.

In summary, using murine model of mandibular defect, we highlight the critical role of NLRP3 in regulating mandibular healing. Our study identifies that UCHL5 interacts with NLRP3 in M-MSCs, which results in the NLRP3 inflammasome activation and inhibits osteoblastic differentiation. This study adds a new insight into understanding the activation of inflammasomes in nonimmune cells. Overall, our findings suggest that a UCHL5-dependent mechanism that controls NLRP3 inflammasome activation might be a potential therapeutic target to mandible skeletal tissue repair.

## Supplementary Material

Supplementary figures and table.
