# Supplementary material for: NLRP3 Regulates Mandibular Healing through Interaction with UCHL5 in MSCs
Source: Int J Biol Sci. 2023 Jan 16;19(3):936–49. doi: 10.7150/ijbs.78174 (PMC9910010; doi:10.7150/ijbs.78174)
Supplement: Supplementary file 1 — Supplementary figures and table. [file ijbsv19p0936s1.pdf]

Supplemental figure 1

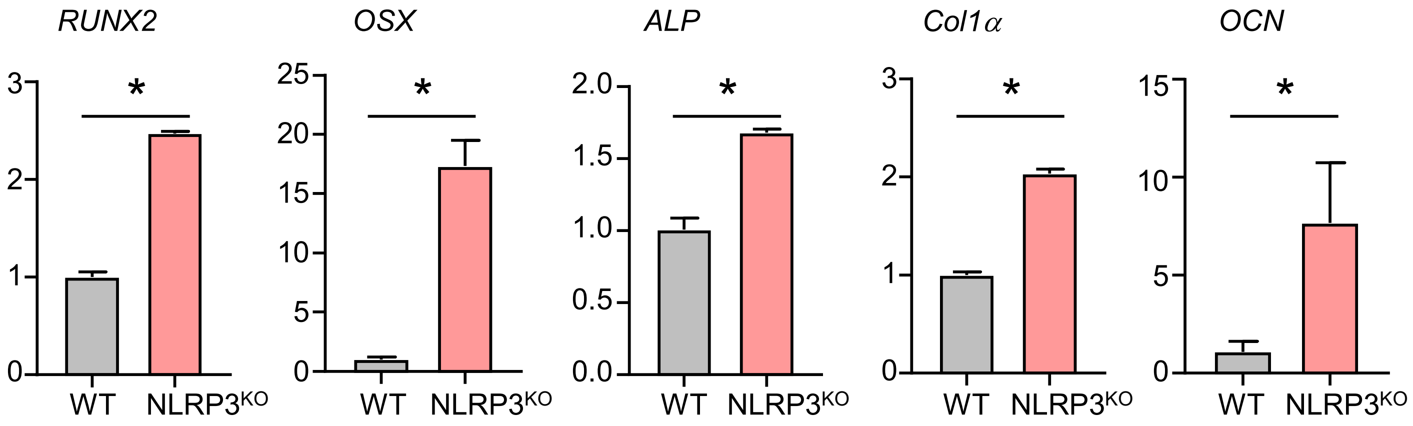

Figure S1. NLRP3 deficiency increases the expression levels of osteoblast-related genes

Mandibular bone marrow cells from 2-month-old NLRP3^{KO} mice and their WT littermates were cultured in osteoblast induction medium for 7 days. The relative gene expression levels of *RUNX2*, *OSX ALP*, *Col1α*, and *OCN* were determined by qPCR. Values are mean ± SEM. Two-tailed unpaired Student's t-test was performed. *$P < 0.05$ in the indicated groups.

Supplemental figure 2

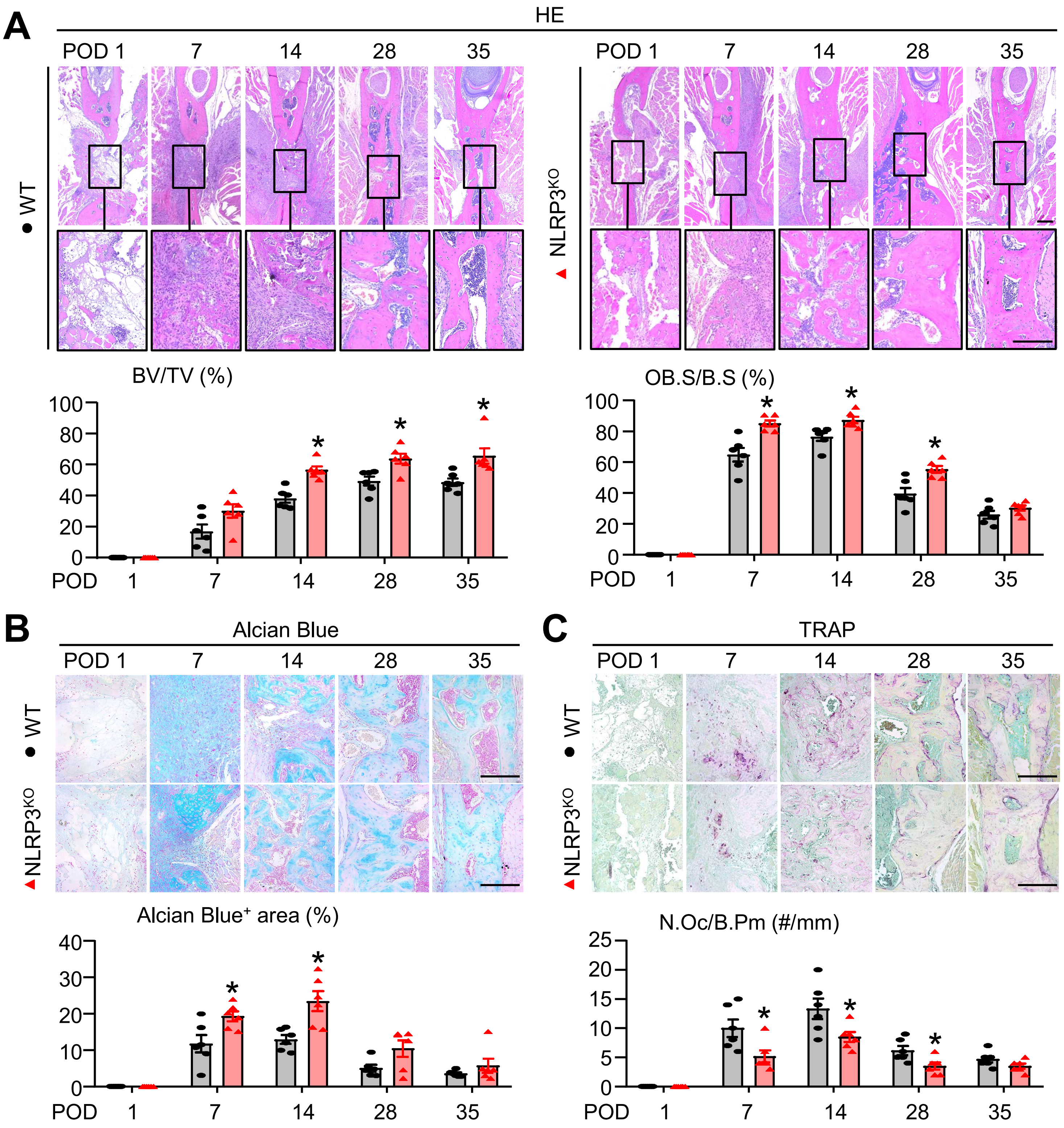

Figure S2. NLRP3 deficiency promotes mandibular healing

Two-month-old WT and NLRP3^{KO} mice received mandibular osteotomy surgery and were sacrificed at various time points during healing, N=6. (A) Representative images of H&E-stained paraffin sections. BV/TV (%) and surface of ALP-positive osteoblasts relative to bone surface (OB.S/B.S) were determined. (B) Representative images of AB-stained paraffin sections. AB-positive area were determined. (C) Representative images of TRAP-stained paraffin sections. Number of TRAP-positive osteoclasts relative to bone surface (#/mm) were determined. Scale bars, 200 μm. Values are mean ± SEM. Two-tailed unpaired Student's t-test was performed. *$P < 0.05$ vs. WT at the same time point.

Supplemental figure 3

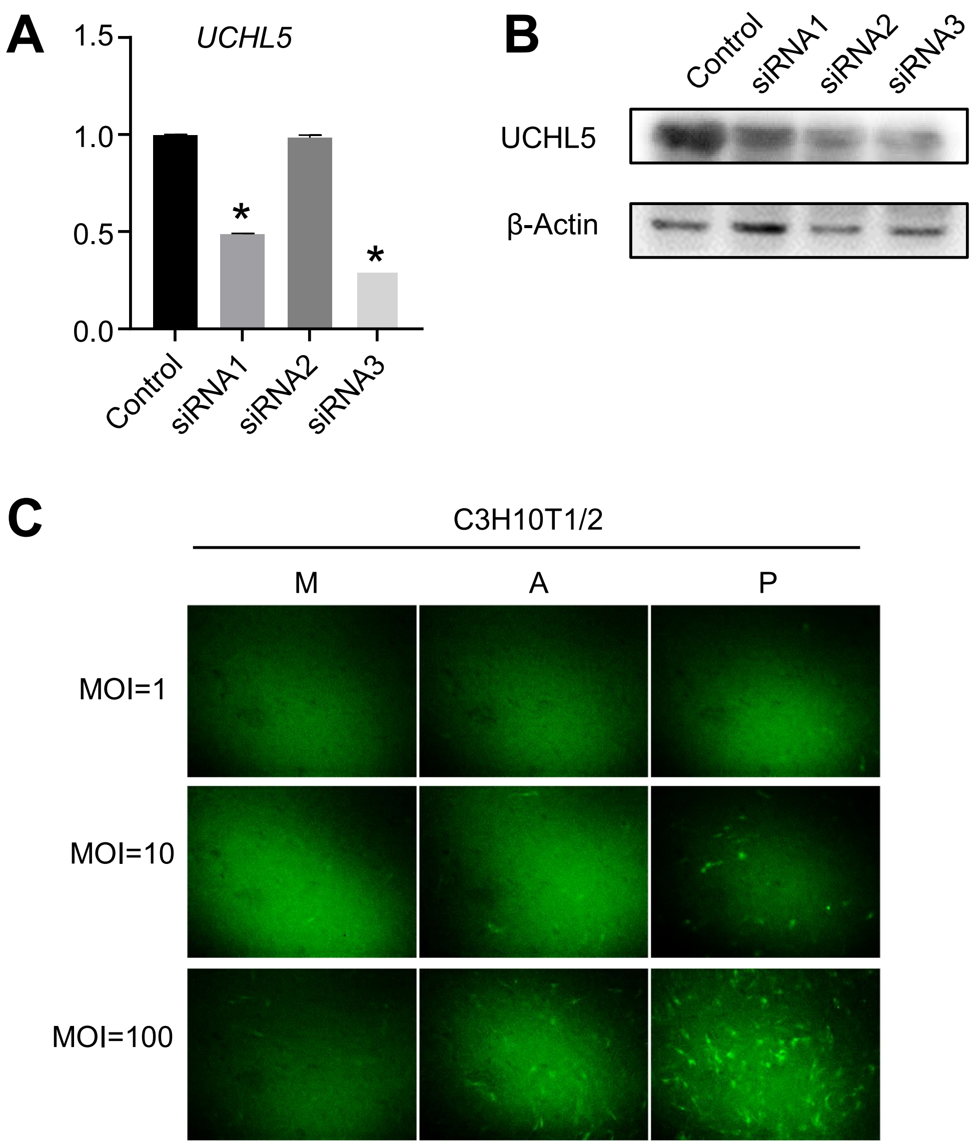

Figure S3. Efficiency testing with different transfection reagents

(A-B) C3H10T1/2 cell lines were cultured and transfected with small interfering negative control RNA (si-NC) or specific UCHL5 small interfering RNA (si-UCHL5), respectively. Transfection efficiency was measured 48 h post-transfection using qPCR (A) and Western blot (B). (C) Lentiviral infection efficiency in cells was determined with the lentiviruses expressing GFP (green-fluorescent protein) 24 h after infection. HitransG A or HitransG P was added to promote the efficient lentivirus infection of cells.

Supplemental figure 4

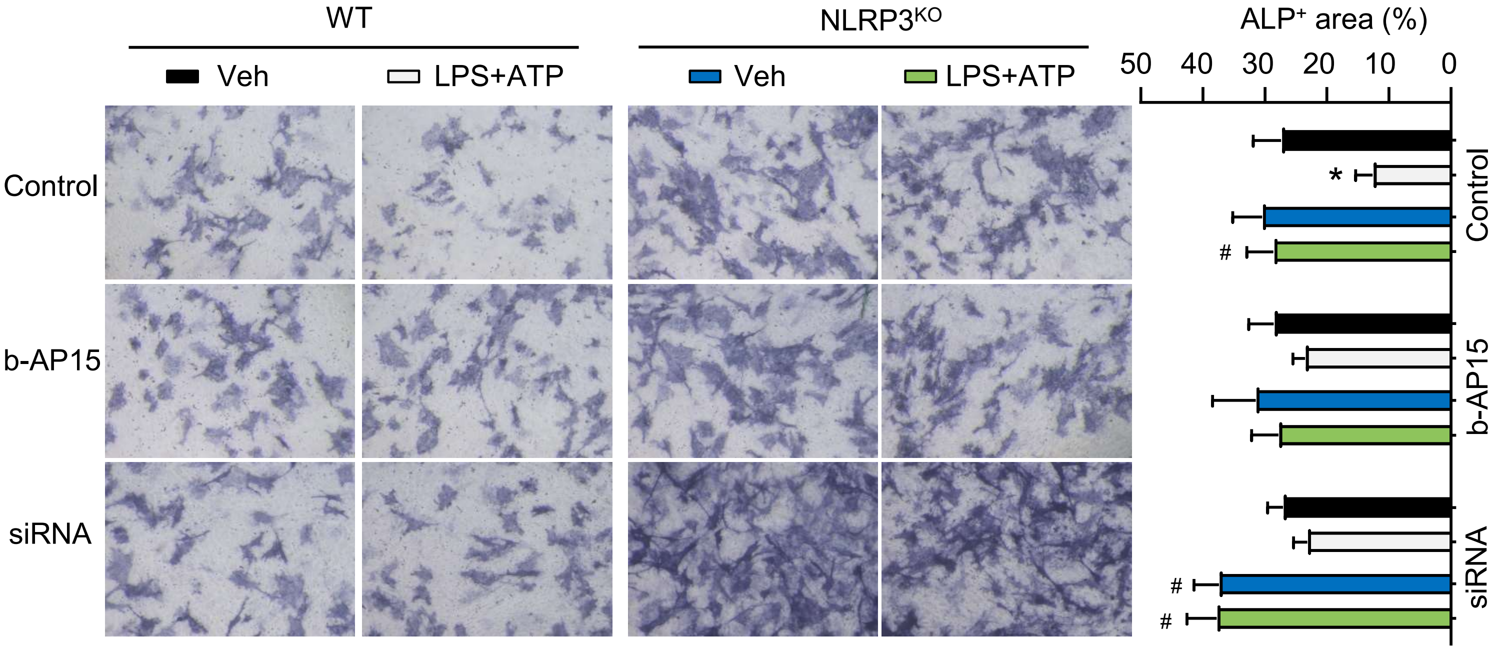

Figure S4. UCHL5 inhibition enhances OB differentiation

M-MSCs from NLRP3^{KO} mice and their WT littermates were treated with b-AP15, or transfected with si-UCHL5. Cells were stimulated with or without 10μg/ml LPS ± 5mM ATP and subjected to ALP staining. ALP^{+} areas were measured. All error bars represent SEM. One-way ANOVA followed by Dunnett's post-hoc multiple comparisons was performed. *P < 0.05 vs. control or WT without treatment, #P < 0.05 vs WT treated with the same reagent.

Supplemental table 1

The sequences of primers used.

Genes	Sequences
GAPDH	F: GGTCGGTGTGAACGGATTTG
	R: ATGAGCCCTTCCACAATG
NLRP3	F: ATTACCCGCCCGAGAAAGG
	R: TCGCAGCAAAGATCCACACAG
IL-1β	F: AACCTGCTGGTGTGTGACGTTC
	R: CAGCACGAGGCTTTTTTGTTGT
Caspase-1	F: ACAAGGCACGGGACCTATG
	R: TCCCAGTCAGTCCTGGAAATG
OCN	F: CTGACCTCACAGATGCCAAG
	R: GTAGCGCCGGAGTCTGTTC
ALP	F: CTTGCTGGTGGAAGGAGGCAGG
	R: CACGTCTTCTCCACCGTGGGTC
Col1α	F: GCTCCTCTTAGGGGCCACT
	R: CCACGTCTCACCATTGGGG
RUNX2	F: ATGCTTCATTCGCCTCACAAA
	R: GCACTCACTGACTCGGTTGG
OSX	F: ATGGCGTCCTCTCTGCTTG
	R: TGAAAGGTCAGCGTATGGCTT
